# Reporting health research translation and impact in the curriculum vitae: a survey

**DOI:** 10.1186/s43058-020-00021-9

**Published:** 2020-03-03

**Authors:** L. Boland, L. Brosseau, S. Caspar, I. D. Graham, A. M. Hutchinson, A. Kothari, K. McNamara, E. McInnes, M. Angel, D. Stacey

**Affiliations:** 1 Ottawa Hospital Research Institute, 501 Smyth Road, Ottawa, ON K1H 8L6 Canada; 2 School of Health Studies, Western University, 1151 Richmond Street, London, ON N6A 3K7 Canada; 3 School of Rehabilitation Sciences, University of Ottawa, Ottawa, ON K1H 8M5 Canada; 4 Faculty of Health Sciences-Therapeutic Recreation Program, University of Lethbridge, 4401 University Drive, Lethbridge, AB T1K 3M4 Canada; 5 School of Epidemiology and Public Health Faculty of Medicine, University of Ottawa, 307D-600 Peter Morand Cresent, Ottawa, ON K1G 5Z3 Canada; 6 School of Nursing and Midwifery and Centre for Quality and Patient Safety Research, Institute for Health Transformation, Deakin University, Geelong, VIC Australia; 7 Monash Health, Clayton, VIC Australia; 8 School of Medicine, Deakin University, 221 Burwood Highway, Burwood, VIC 3125 Australia; 9 Nursing Research Institute St Vincent’s Health Network Sydney, St Vincent’s Hospital Melbourne & Australian Catholic University, Daniel Mannix Building, Australian Catholic University Fitzroy, Fitzroy, VIC Australia; 10 School of Nursing, University of Ottawa, 451 Smyth Road, Ottawa, ON K1H 8M5 Canada

**Keywords:** Research translation, Research activities, Curriculum vitae, Health researchers, Reporting, Impact

## Abstract

**Background:**

Increasingly, health researchers must demonstrate the impact and real-life applications of their research. We investigated how health researchers with expertise in knowledge translation report research translation activities and impact on their curriculum vitae (CV).

**Methods:**

We conducted a cross-sectional survey of health researchers with expertise in knowledge translation as we anticipated best practices in CV reporting from this specialized group. Our survey asked participants about their reporting of research translation and impact activities on their CVs, intention to report, and barriers and facilitators to reporting such activities on their CVs. We calculated univariate descriptive statistics for all quantitative data. Linear regression models determined predictors of researchers’ intention to report research translation and impact activities on their CVs. We analyzed open-ended qualitative responses using content analysis.

**Results:**

One hundred and fifty-three health researchers responded to the survey (response rate = 29%). Most respondents were Canadian, were female, and had a doctoral degree. Eighty-two percent indicated they reported at least one research translation and/or impact indicator on their CVs. Of those, health researchers commonly reported the following: advisory/regulatory committee membership related to research program (83%), research translation award(s) (61%), and academic performance assessments (59%). Researchers least commonly indicated the following: citation metric scores (31%), summaries of impact (21%), and requests to use research materials and/or products (19%). Fewer than half of the health researchers intended to report knowledge translation (43%) and impact (33%) on their CVs. Strong beliefs about capabilities and consequences of reporting research translation and/or impact were significant predictors of intention. Main barriers were as follows: CV templates do not include research translation and impact activities, participants perceived employers do not value research translation and impact activities, and lack of metrics to evaluate research translation and impact. Ninety-six percent were unaware of a CV template formatted to include research translation and/or impact reporting.

**Conclusions:**

Knowledge translation and impact indicators on the CV are inconsistently reported by our sample of health researchers. Modifiable barriers should be addressed to support more consistent reporting of such activities, including providing a CV template that includes research translation and impact as well as clear metrics to quantify them.

Contributions to the literature

- Health researchers are increasingly asked to demonstrate the application of their research findings in clinical practice and/or policy. The curriculum vitae (CV) is one means of recording and communicating such activities.
- Our findings show that knowledge translation and impact indicators on the CV are inconsistently reported and health researchers face numerous, but potentially modifiable, barriers to reporting research translation and/or impact activities on their CVs.
- Health researchers suggest that a CV template that includes research translation and impact would facilitate meeting academe’s expectations for demonstrating and reporting such activities.

## Background

For the academic researcher, the curriculum vitae (CV) is the main document used to record and communicate accomplishments and impact [1–4]. Academics are typically incentivized to use an “output” paradigm to communicate via their CVs, indicating the success of actions that are evaluated against the individual’s capacity to produce knowledge [5]. Rewarded academic outputs have traditionally focused on grant funding received, invited national/international presentations, and peer-reviewed publications [6–8]. Although these outputs are evaluated for tenure and promotion, they do not necessarily indicate researchers’ impact on practice or policy [9]. Given an estimated $200 billion of research funds was spent on research for which research findings were not applied [10, 11], there is increased attention on activities indicating research translation and impact [12].

Research translation and impact activities aim to close the research-practice gap, leading to better patient and health system outcomes [13, 14]. This includes the diffusion, dissemination, and application of evidence such that researchers have made societal contributions beyond academia (e.g., economic, cultural, policy, services). A systematic mapping of knowledge translation strategies and structures by organizations (e.g., large research producers, intermediary agencies, major funders) found eight activity archetypes: producing knowledge, brokering, intermediation, advocating evidence use, researching practice, fostering networks, and advancing knowledge mobilization [15]. Such research translation and impact activities are time-consuming; thus, it is essential that they are recognized. Academic research establishments (e.g., universities, funders, researchers) should be accountable for demonstrating the research translation and impact of funded research [16, 17]. If impact and translation are not well documented, or if researchers feel challenged with its documentation, adverse consequences may follow. For example, it may reduce the influence of one’s translation efforts and impact on subsequent decisions about promotions, grant applications, and other awards. This in turn might promote continued reliance and emphasis on traditional measures of output (e.g., publications and grants) as the core currency and purpose of research.

Accountability for research translation and impact is only beginning to be recognized, and little is known about how health researchers document impact and research translation on their CVs. The purpose of this study was to investigate health researchers’ practices for, and barriers to, reporting research translation and impact activities on their CVs.

## Methods

### Design

We conducted a cross-sectional survey of an international cohort of health researchers. We received Research Board Ethics approval from the University of Ottawa (#H12-15-01), and participants provided informed consent. We followed the STROBE reporting guidelines [18].

### Participants and setting

We recruited a convenience sample of health researchers with expertise in knowledge translation because we anticipated best practices in CV reporting from this specialized group. Health researchers were recruited from the 2015 Knowledge Utilization Colloquium invitee list and those belonging to the Collaborative Health Improvement Partnerships (CHIPs) group of the Canadian Association of Health Services and Policy Research (CAHSPR). Health researchers were defined as individuals who were actively conducting health research. Knowledge Utilization Colloquium attendees included an international group of multidisciplinary academics, researchers, knowledge users, and graduate students.^1^ This meeting aims to advance the science of knowledge utilization in a focused and strategic approach, leading to concrete outputs that extend conceptual and theoretical development and ultimately improve practice in knowledge utilization. The CHIPs group aims to advance and support science underlying integrated knowledge translation as well as to build collaborations between knowledge users and researchers (website: https://www.cahspr.ca/en/themegroups/chips). These two groups were specifically chosen given their research is focused on application of research findings to inform practice, education, and health policy.

### Data collection procedures

We built an online survey using Fluid Surveys TM software and administered it using Dillman’s online survey approach [19]. In 2016, we sent personalized emails to potential participants (*n* = 522) that included an introductory letter, request to participate, and the link to the survey. Non-respondents received three reminder emails at scheduled intervals. Surveys were coded with a unique identifier to ensure no duplicate responses and to protect personal information.

### Data collection tool

Our survey consisted of five sections: (1) demographic questions, (2) a list of potential research translation and impact activities that could be included on a CV, (3) an assessment of intention and related factors influencing reporting translation and impact activities on their CVs based on the Continuing Professional Development Reaction Questionnaire [20], and (4) open-ended questions to identify barriers, facilitators, and general comments. The list of potential research translation and impact activities were generated based on discussions in an Open Space session at the 2015 Knowledge Utilization Colloquium and kept as broad and inclusive as possible. Participants were also asked if they were aware of a template for reporting these types of activities on their CVs.

The Continuing Professional Development Reaction Questionnaire (CPDRQ) is based on the theory of planned behavior and is appropriate for evaluating factors influencing intention to change practice [20]. The instrument includes 12 items that can be adapted to different types of behaviors (e.g., reporting research translation and impact activities on CVs). The questions, using a 7-point Likert scale, evaluate five contructs: intention, beliefs about capabilities, beliefs about consequences, moral norm, and social influences. The CPDRQ’s test-retest reliability was moderate with weighted kappa values between 0.4 and 0.6. Cronbach’s alpha coefficients for the constructs varied from 0.77 to 0.85 [20]. Exploratory factor analysis showed the presence of five constructs, with the proportion of variance explained by each factor greater than 5% [20]. The CDPRQ was also shown to be acceptable to health providers, responsive to, and predictive of subsequent behavior change [21].

### Outcomes

Our primary outcome was health researchers’ reporting of research translation and impact activities on their CVs. Our secondary outcomes were intention to report research translation and impact on the CV, predictors of intentions, and barriers and facilitators to reporting. We defined *research translation activities* as the diffusion, dissemination, and application of knowledge that researchers undertake once the findings from a study are available [13]. *Impact* is the demonstrable contribution that research makes to the economy and the society, beyond academic contributions (e.g., publications and presentations) [22]. Indicators of impact can be instrumental (e.g., development or changes in policy, practice, services, legislation, behavior), conceptual (e.g., contribute to understanding of policy issues, reframing debates), and capacity building (e.g., technical and personal skill development).

### Analysis

Raw survey data were exported from Fluid Survey TM and transferred to Microsoft Excel (Microsoft Corporation, Redmond, WA, USA, 2004) and Statistical Analysis Software for Windows (version 9.4: SAS, Cary, NC, USA, 2019). We calculated univariate descriptive statistics for all quantitative data, including means and standard deviations for the 7-point Likert scores of factors influencing health researchers reporting of research translation and impact. To calculate intention to report research translation and impact activities on CVs, we assigned a construct score for the CDPRQ by calculating the mean and standard deviation of items within each construct. Associations between theoretical constructs and intention to report research translation and impact activities on CVs were calculated using univariate (unadjusted) and multivariable (adjusted) ordinary least squares linear regression models. We calculated a coefficient of determination (*R*^2^) to determine the proportion of variance between the theoretical constructs and intention to report research translation and impact activities on CVs.

Open-ended survey responses were analyzed by two reviewers independently (LB and MA) using content analysis involving five steps: reading the data in its entirety, developing codes to reflect the data, coding the data, a second review of the data, and establishing consensus between the reviewers [23].

### Missing data

Descriptive data were analyzed using a complete case approach. Missing data for inferential analyses of the CDPRQ responses were handled using multiple imputation. As these responses were continuous variables, we used PROC MI and PROC MIANALYZE to perform multiple imputation of missing responses based on fully conditional specification regression methods. We created five multiple imputed datasets, with imputation informed by responses of non-missing items from the CDPRQ.

## Results

### Participants

**Fig. 1:**
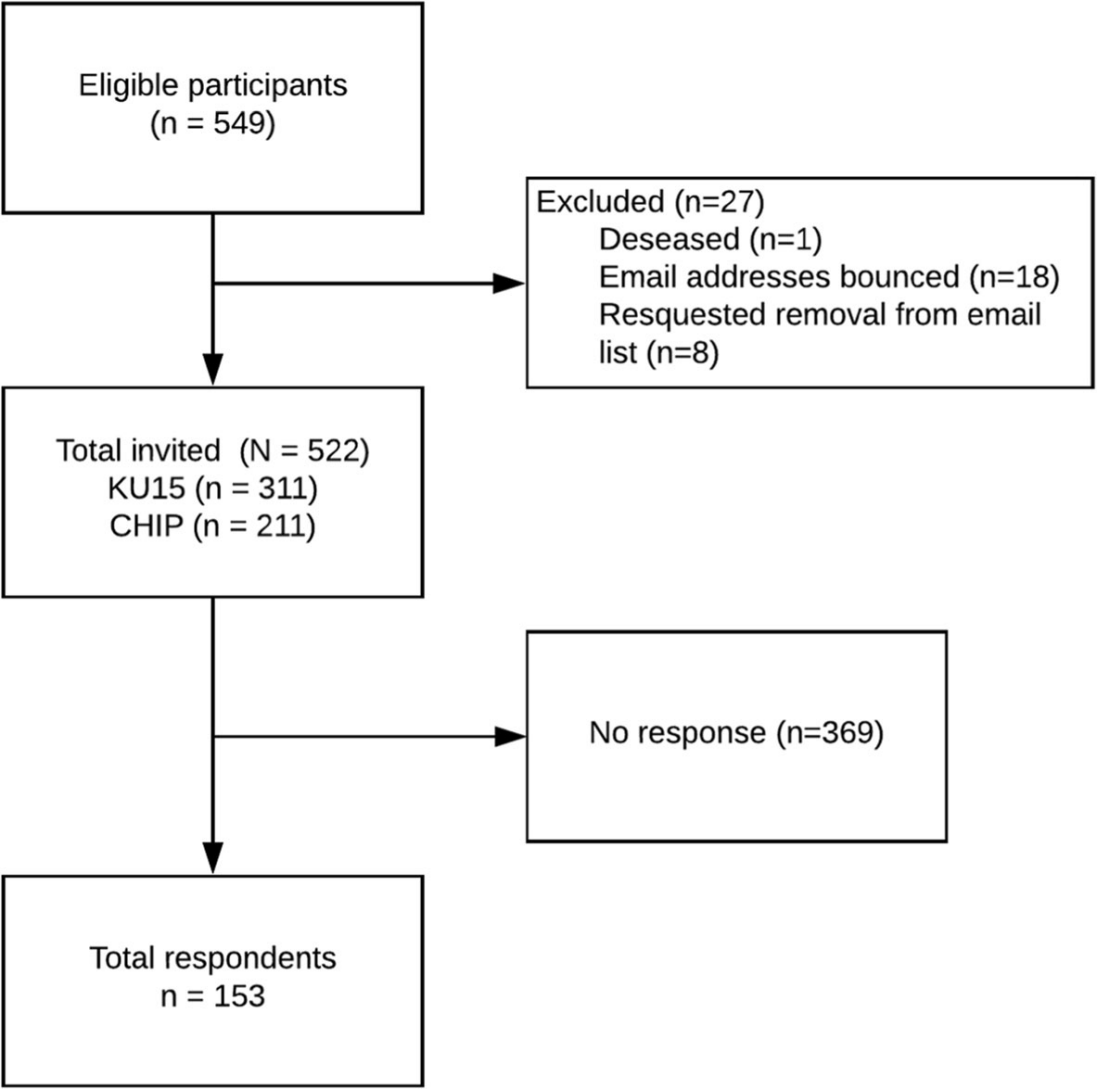
Participant flow

Of the 522 invited health researchers, 153 responded to the survey with a response rate of 29% (Fig. 1). Most respondents were Canadian (39%), were female (83%), and had a doctoral degree (81%). Most were early (22%), mid (29%), or late (27%) career professors. The mean respondent age was 49 years (Table 1).

**Table 1:** Participant characteristics

Characteristics		Frequency n (%)
Age (*n* = 114)	Age, mean (SD), range	49 (*SD* 9.8) (26 - 66)
Sex (*n* = 120)	Female	100 (83%)
Countries (*n* = 127)	Canada	60 (47%)
	Australia	22 (17%)
	Sweden	14 (11%)
	UK	13 (10%)
	USA	8 (6%)
	Other (Netherlands, Vietnam, Ireland, Saudi Arabia, Norway, Japan)	10 (8%)
Highest level of education (*n* = 118)	Doctoral degree	95 (81%)
	Master’s university degree	19 (16%)
	Undergraduate degree	4 (3%)
Position (*n* = 119)	Senior-researcher/Professor	32 (27%)
	Mid-researcher / Associate Professor	35 (29%)
	Early researcher /Assistant Professor	26 (22%)
	Doctoral student/candidate	12 (10%)
	Clinician-scientist	2 (2%)
	Other (Knowledge user, Master’s student, policy administrator, implementation scientist, consultant, administration, post-doctoral fellow)	12 (10%)
Time in current position (*n* = 120)	< 1 year	11(9%)
	1-2 years	24 (20%)
	3-5 years	36 (30%)
	6-10 years	30 (25%)
	11- 20 years	10 (8%)
	> 20 years	9 (8%)
Disciplinary background (*n* = 152)	Nursing	54 (36%)
	Medicine	16 (11%)
	Rehabilitation (occupational therapy, physiotherapy, speech-language pathology, audiology)	8 (5%)
	Sociology	6 (4%)
	Health research	4 (3%)
	Health administration	4 (3%)
	Epidemiology	4 (3%)
	Psychology	3 (2%)
	Other allied health professionals (public health, pharmacy, chiropractic, nutrition)	7 (5%)
	Other (engineering, communication, chemistry, behavioral sciences, general health sciences, administration)	42 (30%)
	Preferred to not report	4 (4%)

### Reporting of research translation and impact activities

Of the 153 participants, 126 (82%) reported at least 1 research translation and/or impact indicator on their CVs. Most commonly, health researchers reported membership on advisory/regulatory committees related to their research (83%) (e.g., hospital Patient Experience Committee), awards and/or recognitions for research translation activities (61%) (e.g., Knowledge to Action Grants), others’ assessments of one’s academic performance (59%) (e.g., promotion, contract renewal), publications targeting specific audiences/professions (56%) (e.g., National Institute for Health and Care Excellence (NICE) policy and guidance reports), and training provided to knowledge users (55%) (e.g., online training programs) (Table 2). Health researchers least commonly reported the following on their CVs: citation metrics of research impact (31%) (e.g., H-index—from Scopus/Web of Science/Google Scholar), summaries of impact (21%) (e.g., 1 page summarizing various research translation and impact activities), and requests for permission received to use materials and/or products resulting from research (19%) (e.g., measurement instruments, knowledge tools such as patient decision aids). Of the 124 who responded to this question, 119 (96%) were unaware of a CV template or guidelines that provide a structured format for reporting research translation activities and/or indicators of impact.

**Table 2:** Health researchers’ reporting of research translation and impact activities on their CVs

Research translation and/or impact activities		Yes n (%)	No n (%)	NA n (%)
Citation metrics	H-index – reported from Scopus, Web of Science, Google Scholar (*n* = 123)	25 (20%)	85 (69%)	13 (11%)
	ResearchGate score (*n* = 124)	7 (6%)	103 (83%)	14 (11%)
	Research cited in policy documents or guidelines (*n* = 123)	26 (21%)	73 (59%)	24 (20%)
	Highly accessed publications (*n* = 124)	35 (28%)	70 (56%)	19 (15%)
	Research cited in journal commentaries (*n* = 123)	10 (8%)	79 (64%)	34 (28%)
	Research cited blogs or websites (*n* = 124)	9 (7%)	90 (73%)	25 (20%)
	Book chapters informed by your research (*n* = 123)	42 (34%)	53 (43%)	28 (23%)
	Publications targeting specific audiences/professions (*n* = 123)	69 (56%)	42 (34%)	12 (10%)
Reporting of products and materials results from research	Products and/or materials designed to translate research into practice or policy (*n* = 123)	53 (43%)	51 (42%)	19 (15%)
	Online downloads or hits from products/materials developed from research (*n* = 124)	14 (11%)	89 (72%)	21 (17%)
	Permission/requests to use products/material (*n* = 124)	6 (5%)	100 (81%)	18 (15%)
	Integration of research program materials into services within a community (*n* = 124)	19 (15%)	76 (61%)	29 (23%)
Training	Training provided to knowledge users (*n* = 123)	67 (55%)	43 (35%)	13 (11%)
	Course Content or Curriculum (including online training modules) informed by your research program (*n* = 121)	38 (31%)	64 (53%)	19 (16%)
Online and media exposure	Website for research program (*n* = 124)	30 (24%)	77 (62%)	17 (13%)
	Blogs that discuss your research (*n* = 124)	7 (6%)	74 (60%)	43 (35%)
	News sources that discuss your research (*n* = 134)	32 (26%)	66 (53%)	26 (21%)
	Twitter followers (*n* = 120)	4 (3%)	91 (76%)	25 (21%)
Meetings and committees	Invitations to meetings to disseminate research (*n* = 123)	71 (57%)	42 (34%)	10 (8%)
	List memberships on advisory committees/ boards/ regulatory committees (*n* = 122)	102 (83%)	12 (10%)	8 (7%)
Consultancy/ collaboration	Memberships and/or contributions to clinical practice guideline development teams (n=121)	49 (41%)	26 (21%)	46 (38%)
	Health services contracts for projects focused on changing knowledge users practice (*n* = 122)	32 (26%)	58 (48%)	32 (26%)
	Consultancies (*n* = 121)	47 (38%)	40 (33%)	34 (28%)
	Flag knowledge users on publications (*n* = 120)	30 (25%)	71 (59%)	19 (16%)
	Flag knowledge users on grants (*n* = 120)	35 (29%)	66 (55%)	19 (16%)
Use of stories	Stories of impact from knowledge users (*n* = 124)	8 (7%)	92 (74%)	24 (19%)
	Stories of impact by you (health researcher) (*n* = 118)	9 (7%)	89 (75%)	21 (18%)
Awards	Award or other formal recognition for research translation activities (*n* = 124)	76 (61%)	14 (11%)	34 (27%)
Impact	Summary statement/bullet points of impact (*n* = 123)	12 (10%)	97 (79%)	14 (11%)
	Impact for research of graduate students that you supervised (*n* = 123)	14 (11%)	77 (63%)	32 (26%)
	Elected membership on a society for which membership requires demonstration of research impact (*n* = 120)	22 (18%)	38 (32%)	60 (50%)
Other	Assessments of your academic performance (*n*=124)	48 (39%)	65 (52%)	11 (9%)
	Are you aware of any CV template or guidelines that provide a structured format for reporting research translation activities and/or indicators of impact? (*n* = 124)	5 (4%)	119 (96%)	0

*NA* not applicable

### Intention to report research translation and impact activities on CVs

Of the respondents, 52/121 (43%) indicated they intended to report research translation and 38/115 (33%) indicated they intended to report impact activities on their CVs. The CPDRQ rating for intention to report research translation and impact activities on their CVs was 4.6 out of 7.0 (Table 3). Unadjusted associations showed that all theoretical constructs, except social influence, were significantly associated with intention to report research translation and impact activities on their CVs. However, adjusted analysis revealed that only beliefs about capabilities and beliefs about consequences were significant predictors of intention to report research translation and/or impact on the CV. Overall, inclusion of all constructs accounted for approximately 36% of the total variation in intention (*R*^2^ = 0.36).

**Table 3:** Association between theoretical constructs with health researchers’ intention to report research translation and impact activities on their CVs

Construct	Score^†^ (mean ± SD)	Univariate regression coefficient	95% CI	Multivariate regression coefficient	95% CI
Intention (*n* = 115)	4.6 (1.8)	–	–	–	–
Social influence (*n* = 51)	3.1 (1.7)	0.24	− 0.02–0.49	0.01	− 0.23–0.25
Beliefs about capabilities (*n* = 112)	4.8 (1.4)	0.56*	0.35–0.78	0.40*	0.17–0.63
Beliefs about consequences (*n* = 113)	5.9 (1.2)	0.69*	0.46–0.92	0.17*	0.19–0.78
Moral norm (*n* = 110)	5.1 (1.4)	0.55*	0.33–0.77	0.48	− 0.19–0.51

*Statistically significant finding at the *P* < 0.01 level

^†^Score range 1 to 7

### Barriers and facilitators to reporting research translation and impact activities

For the quantitative questions asking participants to rate the strength of the barriers/facilitator (1 low to 7 high), the main barriers were (1) lack of time to report research translation and/or impact, (2) lack of support for health researcher’s organization, and (3) access to resources to support reporting of research translation and impact on their CVs (Table 4). The highest rated facilitators were additional training about research translation and impact reporting and a CV template that included research translation and/or impact activities.

**Table 4:** Barriers and facilitators of reporting of research translation and/or impact activities on CVs based on rated survey questions (quantitative)

Stem statements evaluating factors influencing researchers reporting research translation and/or impact activities on the CV	Score (1 = strongly disagree to 7 = strongly agree), M (SD)
I have time to report research translation and/or impact activities (*n* = 116)	3.8/7 (1.7)
My organization supports me in reporting research translation and/or impact activities (*n* = 114)	3.9/7 (1.9)
I have access to resources supporting them in reporting research translation and/or impact activities (*n* = 113)	4.2/7 (1.8)
I think additional training would help them in reporting research translation and/or impact activities (*n* = 114)	5.2/7 (1.8)
I think a CV template that included research translation and/or impact activities would help my reporting (*n* = 118)	6.3/7 (1.2)

Barriers to reporting research translation and impact that emerged on the open-ended survey question included the perception that employers, funders, and/or colleagues do not value or expect reporting of research translation and impact activities on the CV; concerns that others will perceive reporting of research translation and impact as an attempt to embellish accomplishments and/or bolster the CV; lack of valid and reliable metrics and/or indicators for reporting research translation and impact activities; and space constraints on the CV (Table 5). Open question facilitators to reporting included self-efficacy, positive attitudes, and the option to use unstructured CVs.

**Table 5:** Barriers and facilitators of reporting of research translation and/or impact activities on the CV based on open-ended question (qualitative)

Factors influencing reporting research translation and/or impact activities on CVs	B	F
Extent that researchers had access to resources supporting them in reporting research translation and/or impact activities	√	√
Extent that researchers had time to report research translation and/or impact activities	√	√
Researchers lacked the knowledge, awareness, skills to report research translation and/or impact	√	
Perception that organization, employers, funders, and/or colleagues do not support or value reporting research translation and/or impact activities	√	
Concern that others will think reporting research translation and/or impact activities is an attempt to inflate achievement	√	
Valid and reliable metrics and indicators of research translation and/or impact activities are lacking	√	
Space constraints on the CV limits reporting of research translation and/or impact activities	√	
Extent that researchers thought training would help them in reporting research translation and/or impact activities		√
Extent that researchers thought a template that included research translation and/or impact activities would help their reporting		√
Perception that adding research translation and/or impact activities to the CV would be easy		√
Awards related to research translation and/or impact activities		√
Positive attitudes about including research translation and/or impact activities		√
Unstructured and/or non-academic CVs		√
If it was common practice (social norm)		√
If it was reinforced by others		√
Self-efficacy		√

*B* barrier, *F* facilitator

## Discussion

Health researchers are increasingly required to show evidence of translation of their research findings to demonstrate real-world impact [9, 24]. As such, it is important that health researchers capitalize on opportunities to document and communicate the impact of their research. Our study used a survey to evaluate health researchers’ current reporting research translation and impact activities on their CVs, their intention to report, and barriers and facilitators influencing reporting these activities on their CVs. Health researchers most commonly reported membership on advisory/regulatory committees related to their research and least commonly reported citation metric scores (e.g., ResearchGate Score). Fewer than half of the health researchers intended to report research translation and impact on their CVs, with beliefs about capabilities and beliefs about consequences being significant predictors of intention. Despite this intention, health researchers faced several barriers to reporting translation and impact. Our findings lead us to the following observations.

First, although most health researchers reported at least one research translation and/or impact activity, few provided a comprehensive account of the application of their research to practice or policy, suggesting that reporting practices could be improved. Furthermore, there were inconsistencies in what was reported on the CV across respondents. A recent systematic review evaluated the reporting quality of research translation and impact case studies and found a lack of consistent reporting approaches, including few demonstrated evidence for the reported impact [8]. Our study also demonstrated health researchers are inconsistently reporting research translation and impact on their CVs, and struggling to measure and quantify these activities. Based on this study, we adapted our survey questions into a guide for what can be reported with examples (Appendix A).

Second, our study could have increased participants’ awareness of reportable research translation and impact activities and their intention to report. Predictors of intention included health researchers’ beliefs about their capabilities and beliefs about consequences of reporting. These constructs align with qualitative statements that health researchers lacked skills to report (capabilities), perceived that others did not value such reporting on CVs (consequences), and that reporting these activities could be negatively perceived as inflating one’s CV (consequences). However, health researchers wanted training to improve their research translation and impact skills (enhance capabilities). These findings suggest that interventions to promote the reporting of research translation and impact should include strategies that improve health researchers’ beliefs about their capabilities and the potential positive consequences of such reporting. For example, interventions could build knowledge and skill regarding reportable research translation and impact activities. Health researchers’ beliefs about consequences could be leveraged with strategies that incentivize and reinforce the behavior, such as validating research translation and impact metrics and having universities more systematically consider research translation and impact for tenure and promotion [25, 26].

Third, although some intend to report research translation and/or impact on their CVs, health researchers indicated several barriers to doing so. Engaging in, monitoring, and reporting research translation activities were deemed time-consuming, requiring energy, resources, and funds, all of which are commodities that health researchers invariably feel are in short supply. This raises questions such as whether organizations *should* support researchers to improve this reporting, and if the reported lack of time actually indicates a lack of prioritization. Certainly reporting additional items might be seen simply as not worthwhile if there are no perceived personal benefits (i.e., consequences) or organizational imperatives around documentation of translation and impact. Furthermore, documentation of impact and translation is more labor intensive than documentation of traditional measures of research performance (e.g., publications and grants), and may warrant assistance from organizations (i.e., improve capabilities/capacity).

A recent systematic review found that accurately and transparently measuring research translation and impact is challenging [8]. In fact, many knowledge translation measures (e.g. uptake by clinicians in other health systems or use of findings for educational purposes) are often poorly documented and difficult to verify compared what is traditionally reported (e.g., publications, grants, and awards are easy to verify through databases and online services). And, it often takes months or years to have an impact, which is more likely to occur in combination with a larger body of work. The Leiden Manifesto discusses the pitfalls and biases of traditional metrics of impact and principles to improve assessment of impact (e.g., include quantitative and qualitative information, be transparent about the assessment process, scrutinize and update indicators routinely) [27]. In our study, health researchers were unsure what research translation and impact activities to track and how to accurately provide evidence of impact. This reality, combined with the disproportionate emphasis the academe places on published findings in high-impact, peer-reviewed journals, seems to contribute to health researchers placing the tracking of their research translation and impact activities lower on their priority lists. As such, the development and validation of metrics to quantify research translation and impact should be a priority.

Health researchers’ ability to report research translation and impact is often influenced by systematic structures, such as specific CV templates used for different agencies and/or purposes (e.g., funding agencies and tenure and promotion). Traditionally, existing research structures do not readily support monitoring and evaluation of research translation and impact [27]. Increased recognition, at societal and academic levels, of research impact importance has led some funding bodies and universities to modify the requirements for CV reporting to capture evidence of research translation and impact. Such requirements at institutional levels can strongly influence the culture and practice of reporting. While such measures and tools are emerging^2^, empirical evaluation of their validity and reliably is still lacking [28]. Various organizations internationally have proposed mechanisms and indicators for assessment of research impact. The Bernard Becker Medical Library Project proposes a model for assessment of research impact to track diffusion of research outputs and activities focused on advancing knowledge, clinical implementation, legislation and policy, economic benefit, and community benefits [29], whereas the Canadian Academy of Health Sciences has a list of preferred indicators and metrics of impact categorized by advancing knowledge, research capacity-building, informing decision-making, health-impact, and broad economic and social impacts [17]. Software companies have also begun developing applications to evaluate research impact.^3^ While it is encouraging that some organizations allow for, and indeed require, evidence of impact in reporting templates, such templates may be unnecessarily restrictive, precluding reporting of some forms of evidence of impact. As such, templates need to be able to accommodate various types of information that represent research translation and impact.

Finally, health researchers in our study suggested that templates and guidance documents for research translation reporting are required and could help incentivize and shift research thinking from “outputs” to include “impact.” However, our study suggests that most respondents were unaware of existing CV templates that include research translation and impact. When we asked respondents to share templates, many responded with requests for guidance on how to better report on these activities. For example, national granting agencies, such as the Canadian Institutes of Health Research and the National Institutes of Health, invite researchers to briefly describe their impact but this is limited to including up to five important contributions to science.^4^ A recent scoping review that identified existing research translation frameworks concluded that a gap involves frameworks to optimize research translation and impact [25].

Our study findings should be considered within the context of its limitations and strengths. Our survey had a low response rate (29%), which increases the likelihood of non-response bias. In fact, those interested in reporting knowledge translation and impact activities may have been more likely to participate, and our findings could represent a best-case scenario of research translation and impact reporting on the CV, which could have led to selection bias. Similarly, our results might have been different had we sampled from a broader population of health researchers (i.e., those without expertise in knowledge translation and impact). Another potential bias is social bias (i.e., attributing characteristics about research translation and impact reporting on the CV to all members of the group). However, our study was strengthened by the inclusion of international perspectives and use of a validated measure to evaluate intention and related barriers. Our study is a starting point for a larger multicountry survey of a broad representation of health services researchers to understand how responses may differ across countries.

## Conclusions

Funding agencies are increasingly holding health researchers accountable for demonstrating the value and impact of their publically funded research. The CV is one medium for health researchers to document the practical importance of their research and communicate it to others. Our study suggests that health researchers, who recognize the importance of research translation, are reporting research translation and impact in ways that appear unsystematic and non-structured. This inconsistency can be in part explained by the barriers to reporting research translation and impact, such as unaware of how to report it, whether it is accurate, and whether it is valued by funders and employers. Academe must evolve to better value research translation and support health researchers in adjusting to a shifting research landscape requiring demonstration of impact. As a first step, health researchers who participated in this study suggested that CV templates be expanded to include research translation and impact. Additionally, our study might provide an impetus for funders and academic institutions to recognize and credit research that is impactful and applied to practice or policy.

## Supplementary information

**Additional file 1.** Suggestions of research translation and impact activities to be reported in Academic Curriculum Vitae.

## Data Availability

Individuals interested in accessing the data used in this study are invited to contact the corresponding author.

## Abbreviations

CPDRQ: Continuing Professional Development Reaction Questionnaire
CV: Curriculum vitae

## Footnotes

1 https://www.ualberta.ca/nursing/research/research-units/knowledge-utilization-studies-program/knowledge-utilization-colloquia

2 www.impactstory.org; www.altmetric.com

3 VV–Impact Tracker: https://www.vertigoventures.com/vv-impact-tracker/

4 https://guides.lib.unc.edu/NIH-biosketch
