# Supplementary material for: Reporting health research translation and impact in the curriculum vitae: a survey
Source: Implement Sci Commun. 2020 Mar 3;1:20. doi: 10.1186/s43058-020-00021-9 (PMC7427883; doi:10.1186/s43058-020-00021-9)
Supplement: Supplementary file 1 — Additional file 1. Suggestions of research translation and impact activities to be reported in Academic Curriculum Vitae. [file 43058_2020_21_MOESM1_ESM.docx]

**Appendix A: Suggestions of research translation and impact activities to be reported in Academic Curriculum Vitae**

| Category | Examples | Description | Where to report | Rationale |
| --- | --- | --- | --- | --- |
| Diffusion | Social media used as part of your research program | - Website - Blog site - Twitter handle; Twitter followers - YouTube channel | Add as part of your contact information and/or your social media personal details | To provide other sources of information used to communicate research findings. |
| Dissemination | Invited presentations | - Invitations to meetings to disseminate research to knowledge users to inform policy, education, practice | In the section on invited presentation, indicate the target audience including knowledge users. | To provide evidence that presentations are being given to those who could use the knowledge. |
| Dissemination | Resources/ products | - Resources to translate research into practice or policy (e.g., knowledge tools, interventions, policy briefs, programs, new positions created, etc.) | Add sub-section in publications on products or tools. | To provide evidence of tools and resources produced that facilitate transfer of evidence into practice. |
| Dissemination | Training knowledge users | - Training workshops provided to knowledge users (e.g., health professionals, patients, and/or policy makers) | In a section on workshops given, indicate if knowledge users attended.  Rationale: | To provide evidence of engagement with knowledge users |
| Dissemination, impact and recognition | Other citations of your work | - Reports in media or news written about or to include your research program (circulation/listenership/ market share) - Policy documents, government/health organization reports or clinical practice guidelines that cite your research - Commentaries in journals citing your research - Blogs/websites of others citing your research - Book chapters that cite your work | Add as a sub-section of publications  Rationale: provides evidence of others commenting or using your work  Add a specific category about the influence of your work on other documents.  Rationale: provides evidence that your research is influencing decision making/policy | Article written by others about my research:  The Ottawa Hospital (2015). Decision aids to help patients weigh options and make choices. The Ottawa Hospital newsroom (hospital newsletter distributed to 15,000 subscribers). |
| Dissemination and application | Recognize involvement of knowledge users (i.e., integrated research translation) | - Knowledge users indicated on publications - Knowledge users indicated on grants - Knowledge users indicated on presentations | Add * or other symbol beside the names of knowledge users. | To provide evidence of engagement with knowledge users. Same approach often used to identify graduate students. |
| Application | Funding and projects about knowledge translation | - Projects, contracts, grants focused on changing knowledge users practice | If any are specific to knowledge translation (KT) or impact, then indicate in a bracket (KT Science, KT practice) | To profile specific funding supporting research translation |
| Impact -citation | Bibliometric Measures | - H-index – from Scopus/ Web of Science/Google Scholar   H-index is an author-level metric that attempts to measure both productivity and citation impact of publications. It is based on the most cited papers and number of citations received in other publications. | Report a brief summary of bibliometric measures at the beginning of your publication list and/or summary of impact. | To be an indicator of your work being cited. |
| Impact- citation and recognition | Other publication characteristics | - Publications awarded “highly accessed” and/or editor’s choice by journals - Publications chosen as best article in a journal - Citations | Add as part of the publication description. | To provide evidence of others reading the work or valuing it |
| Impact | Committee membership | - List your memberships on advisory committees/ boards/ regulatory committees - List your memberships and/or contributions to clinical practice guideline development teams | In the section on committees, add a statement about your role as it relates to KT if appropriate | Demonstrates how your research is leading to recognition and influence for decision-making. |
| Impact | Summary on impact | Summary statement/bullet points of indicators of impact from your research that may include:   - Award(s) or other formal recognition for research translation activities - Elected membership to a society for which membership requires demonstration of research impact - Stories of impact by you or knowledge users or others (e.g., changes in practice locally, state-wide, national including de-implementation) - Integration of research program materials into services within a community (e.g., most significant research contributions) - Course content or curriculum informed by your research program | Add a specific section summarizing impact and include indicators of knowledge translation related activities | To provide a brief summary of your CV with indicators of research translation and impact |

NOTE: ResearchGate score was removed because the methods for calculating it are not transparent and there is some question regarding its quality. We defined research translation activities as the diffusion, dissemination, and application of knowledge that researchers undertake once the findings from a study are available [13]. We *used A Glossary for Dissemination and Implementation Research in Health* (Rabin et al., 2008) to guide our definitions of diffusion (passive, untargeted, unplanned, and uncontrolled spread of new interventions/research evidence), dissemination (an active approach of spreading evidence to the target audience via determined channels using planned strategies), and application as the (process of putting to use or integrating evidence-based interventions within a setting). Impact is the demonstrable contribution that research makes to the economy and the society, beyond academic contributions (e.g., publications and presentations) [23].

Rabin BA, Brownson RC, Haire-Joshu D, Dreuter MW, Weaver NL. 2008. A glossary for dissemination and implementation research in health. J Public Health Manag Pract. 14(2):117-123.
